# A regulation-aware architecture for governing AI-generated content: provenance, labeling, privacy, safety, and institutional compliance

**DOI:** 10.3389/frai.2026.1885306

**Published:** 2026-10-21

**Authors:** Iván Miguel García López, José Martín Molina Espinosa, María Soledad Ramírez Montoya, Nicia Guillén Yparrea

**Affiliations:** 1 School of Engineering and Sciences, Tecnologico de Monterrey, Monterrey, Mexico; 2 Benemérita Escuela Normal de Coahuila, Saltillo, Mexico; 3 Institute for the Future of Education, Tecnologico de Monterrey, Monterrey, Mexico

**Keywords:** AI governance frameworks, AI-generated content, content provenance, privacy and safety, public-interest institutions, regulatory compliance, synthetic content labeling, trustworthy AI

## Abstract

**Introduction:**

The rapid adoption of generative AI across multiple modalities has raised concerns about authenticity, labeling, privacy, safety, accountability, and legal compliance in AI-generated content produced within public-interest institutions, particularly in education and public administration. Although prior research has advanced techniques such as watermarking, provenance tracking, and content authentication, these solutions are often developed in isolation, leaving institutions without an integrated governance approach across the content life cycle.

**Methods:**

This paper follows a conceptual architecture design approach combining an integrative literature review with a design-science research logic. Based on this synthesis, the paper introduces a layered, regulation-aware governance framework structured around five interdependent dimensions: provenance and authenticity, labeling and disclosure, privacy and safety safeguards, accountability and compliance controls, and operational governance workflows. The framework conceptualizes synthetic-content governance as a systems-level challenge requiring coordination between technical mechanisms and institutional oversight, specifying how provenance metadata, labeling protocols, privacy-preserving controls, safety filters, audit logs, and human review processes can be operationally combined.

**Results:**

The framework is positioned in relation to established governance instruments—including the NIST AI Risk Management Framework, the OECD AI Principles, the EU AI Act, the UNESCO Recommendation on the Ethics of Artificial Intelligence, and the General Data Protection Regulation—and its layers are mapped to concrete regulatory and standards-based obligations. Its applicability is examined through an illustrative analytical application to documented institutional incidents involving non-consensual synthetic imagery in educational settings.

**Discussion:**

The paper contributes by integrating security, privacy, safety, and regulatory compliance into a unified architecture, translating regulatory expectations into implementable institutional controls, and advancing a public-interest perspective that frames AI-generated content governance as a matter of institutional risk and responsibility. The paper's contribution is thus integrative and operational: it consolidates fragmented and weakly operationalized mechanisms into a single life-cycle framework of governance functions that institutions can adapt across contexts.

## 1 Introduction

The rapid expansion of generative artificial intelligence has transformed the production, circulation, and consumption of digital content across text, image, audio, and video modalities. What was initially perceived as a productivity breakthrough has quickly become a governance challenge, as AI-generated content now affects education, journalism, government, commerce, and public communication at scale (Lorenz, 2023). This shift has intensified concerns over authenticity, manipulation, attribution, and public trust, particularly in contexts where synthetic content can shape institutional decisions or influence socially consequential behavior (Fisher et al., 2024). Recent work has shown that AI-generated content is no longer a marginal technical issue, but a central socio-technical problem involving security, privacy, safety, and accountability.

A central reason for this concern is that the quality of AI-generated content is advancing faster than the governance mechanisms needed to identify, verify, and regulate it. Existing research has documented how generative systems can be used to produce persuasive but misleading outputs, imitate human authorship, and blur the boundaries between authentic and synthetic information (Zhou et al., 2023). In legal and policy terms, this creates pressure for stronger provenance, labeling, and verification mechanisms, while also exposing unresolved tensions related to copyright, data protection, and institutional liability (Novelli et al., 2024). These tensions are especially visible in domains such as education and public administration, where content is not merely expressive but often embedded in decision-support, communication, and compliance processes.

At the same time, the emerging literature suggests that regulation cannot rely exclusively on ex post legal responses or on generic ethical principles. Technical countermeasures such as watermarking, fingerprinting, and labeling are increasingly recognized as necessary, but they remain uneven in robustness, interoperability, and practical impact (Zhao et al., 2025). For example, current studies on AI-generated content labels indicate that labeling alone may have limited effects on credibility and user behavior, which implies that effective governance must combine technical attribution mechanisms with institutional oversight and context-sensitive policy design (Wittenberg et al., 2025). Similarly, recent surveys of watermarking and responsible AI governance highlight that trustworthiness depends on the interaction between technical controls, organizational procedures, and regulatory obligations rather than on a single safeguard.

This gap is particularly relevant for institutions that operate in public-interest environments, including universities, regulatory agencies, and public administrations. In such settings, AI-generated content can influence academic communication, policy interpretation, administrative records, student-facing information, and formal decision-support workflows (Naqvi et al., 2025). Yet most existing work examines either the technical detection of synthetic content or the policy implications of generative AI in isolation, without offering an integrated account of how institutions should govern AI-generated content under simultaneous requirements of security, privacy, safety, and legal compliance (Eisenberg et al., 2025). This fragmentation is increasingly problematic as educational institutions and public organizations adopt generative AI under local policies that remain partial, uneven, or weakly operationalized. The gap addressed here is therefore not the absence of relevant work: substantial technical, legal, and organizational scholarship already treats provenance, labeling, privacy, safety, and accountability. Rather, the gap is one of fragmentation and limited institutional operationalization, because these contributions are developed separately and rarely show how a single institution can combine them into an integrated governance architecture spanning the content life cycle.

Within education, the scope of this paper is deliberately delimited. It does not address generative AI primarily as a pedagogical tool, nor does it focus on student AI literacy, classroom instruction, or academic-integrity detection as ends in themselves. Instead, it concentrates on the institutional governance of AI-generated content: how universities and education authorities manage the provenance, disclosure, privacy, safety, and accountability of synthetic content as it circulates through assessment records, student-facing communication, administrative documentation, guidance systems, and public information. Under this delimitation, education is treated alongside public administration as a public-interest setting in which AI-generated content is embedded in consequential institutional processes rather than circulating only as open web media. This narrower framing makes the analytical scope more precise and clarifies the institutional level at which the proposed framework is intended to operate.

This paper addresses that gap by proposing a regulation-aware governance perspective on AI-generated content, focused on the institutional conditions under which synthetic content can be securely managed, privacy-preservingly handled, safely deployed, and normatively regulated. Rather than treating labeling, provenance, privacy, and accountability as separate topics, the study argues that they should be understood as interdependent dimensions of a unified governance problem (Devagiri, 2025). The main objective of the paper is therefore to develop a conceptual framework for the governance of AI-generated content in education and public administration, with particular attention to labeling, accountability, and institutional compliance (Pinho et al., 2025). Beyond proposing the framework, the paper makes three further contributions that respond to the need for analytical rigor: it positions the framework explicitly in relation to established AI-governance instruments; it maps each framework layer to concrete regulatory and standards-based obligations; and it examines the framework's applicability through an illustrative analytical application to documented institutional incidents. Its main differentiator from adjacent work is that it does not analyze these issues from a purely technical or purely legal angle; instead, it positions AI-generated content governance as a cross-domain problem requiring coordination between advanced technologies, organizational safeguards, and regulation-aware design. Accordingly, the contribution of this paper is integrative and operational rather than foundational: its originality lies not in filling an empty space in the literature but in bringing dispersed technical, legal, and organizational concerns together into a content-specific, life-cycle-oriented governance framework for public-interest institutions.

## 2 Related literature

Research on AI-generated content has expanded rapidly across text, image, audio, and video, with recent surveys showing that the field has moved from model-centric innovation to broader concerns about authenticity, misuse, governance, and societal impact. Comprehensive reviews describe AIGC as a multimodal ecosystem whose risks are not limited to misinformation, but also include attribution failure, copyright uncertainty, manipulation, and trust erosion in institutional settings (Cao et al., 2025). This shift is especially relevant for education and public administration, where synthetic content may be embedded in communication, assessment, records, guidance, or policy support rather than circulating only as open web media (Janumpally et al., 2025). In that sense, the literature increasingly treats AI-generated content as a socio-technical governance problem rather than a purely generative modeling problem.

A first major strand of work focuses on detection, forensics, and content authentication. Recent surveys on AIGC forensics and defenses against AI-generated visual media show that the field has developed rapidly around detector robustness, multimodal benchmarking, and adversarial resilience, but also that a persistent arms race remains between generation quality and forensic capability (Deng et al., 2024). In parallel, watermarking and fingerprinting research has become central for text and LLM-generated content, with surveys emphasizing that watermark robustness, spoofing resistance, and deployment compatibility remain open challenges, especially under paraphrasing, editing, or model transfer scenarios (Yang et al., 2025). These findings suggest that technical authenticity mechanisms are necessary, but not sufficient, because institutional reliability depends not only on whether content can be detected as synthetic, but also on whether provenance can be preserved across real workflows.

A second strand addresses labeling and provenance mechanisms as means to communicate the synthetic status and origin of generated content. User-centered studies indicate that label design matters significantly for how people assess AI-generated media, but they also show that labeling effects are uneven and context-dependent rather than universally corrective (Gamage et al., 2025). At the systems level, provenance-oriented approaches argue that authenticity should be supported by signed manifests, content credentials, and interoperable metadata chains that allow institutions to verify source, transformation history, and authorship claims (Simmons and Winograd, 2024). This literature is highly relevant for regulation-aware design because it reframes provenance not as an optional transparency feature, but as a technical and organizational precondition for enforceable accountability.

A third body of work emphasizes privacy and security risks associated with generative AI and AI-generated content. Surveys on security and privacy in AIGC show that the adoption of generative systems introduces vulnerabilities related to data leakage, prompt injection, model extraction, untrusted outputs, and the secondary exposure of sensitive organizational or personal information (C. Chen et al., 2023). Complementary research on the future of information privacy under AI highlights that opacity, information weaponization, and weak enforcement mechanisms can transform generative systems into vectors of privacy erosion rather than productivity enhancement (Akakpo et al., 2025). These findings are especially important in education and public administration, where generated content may rely on or reveal institutionally sensitive records, thus making privacy-preserving governance inseparable from content governance.

A fourth and increasingly influential strand concerns responsible governance and regulation-aware design. Recent reviews of responsible AI governance argue that effective governance must combine structural, relational, and procedural practices, rather than relying only on high-level ethical principles (Leslie et al., 2023). Legal scholarship on generative AI and the AI Act similarly shows that transparency and compliance obligations are becoming more operational, particularly in relation to copyright, disclosure, and system accountability (Novelli et al., 2024). Taken together, these studies indicate that the governance of AI-generated content requires an integrated approach that links technical controls such as labeling and watermarking with institutional rules, auditability, and legal compliance (Nemecek et al., 2025). However, the literature still remains fragmented: detection work often under-specifies governance, governance work often under-specifies architecture, and privacy work is rarely integrated with provenance and labeling in a unified institutional framework. It is precisely this gap that motivates the present paper.

Alongside this scholarly literature, a layer of formal governance instruments has become directly relevant to AI-generated content. Soft-law and standards-based instruments—including the OECD Recommendation on Artificial Intelligence (OECD, 2019), the UNESCO Recommendation on the Ethics of Artificial Intelligence (UNESCO, 2021), the NIST AI Risk Management Framework (Tabassi, 2023), and the ISO/IEC 42001 management-system standard (International Organization for Standardization, 2023)—articulate principles of transparency, accountability, traceability, and risk management that institutions are increasingly expected to operationalize. Hard-law instruments are also crystallizing: the EU AI Act (European Parliament Council of the European Union, 2024) introduces transparency and disclosure duties for synthetic and manipulated content, alongside record-keeping, risk-management, and oversight obligations for higher-risk uses, while the General Data Protection Regulation (El Ali et al., 2024) constrains how personal data may be processed within generative pipelines. These instruments rarely speak directly to the content life cycle, yet they define the obligations against which institutional AI-generated content governance will be assessed. This reinforces the need for a framework that translates dispersed legal and standards-based expectations into deployable institutional controls, a task taken up in Sections 5.7 and 5.8.

## 3 Methods

This study follows a conceptual architecture design approach that combines an integrative review of recent literature (Snyder, 2019) with a design-science research logic (Hevner et al., 2004; Peffers et al., 2007). The aim of the method was not to conduct an exhaustive systematic review, but to develop a regulation-aware governance architecture capable of translating dispersed technical, organizational, and regulatory concerns into an operational framework for public-interest institutions. An integrative review is appropriate here because the research question requires connecting fragmented bodies of knowledge—detection, provenance, labeling, privacy, and governance—rather than aggregating empirical effect sizes or estimating prevalence. A design-science orientation is equally appropriate because the paper addresses a design-oriented governance problem: institutions do not only need to understand the risks of AI-generated content, but also require structured controls, workflows, and evaluation criteria that can guide implementation.

To support transparency and reproducibility, the literature underpinning the framework was identified through a structured search of academic databases and policy or standards repositories. Searches were carried out across Scopus, Web of Science, IEEE Xplore, the ACM Digital Library, and SpringerLink, complemented by targeted retrieval from authoritative policy and standards sources (EUR-Lex, OECD.AI, UNESCO, and NIST) and, for emerging technical work, from the arXiv and SSRN preprint servers. The search combined five keyword clusters corresponding to the framework dimensions: (i) AI-generated and synthetic content; (ii) provenance, content credentials, watermarking, and fingerprinting; (iii) labeling, disclosure, and transparency; (iv) privacy, security, and data protection in generative AI; and (v) AI governance, responsible AI, compliance, and accountability. Priority was given to peer-reviewed studies, technical surveys, recognized governance frameworks, standards documents, and legal-policy analyses, published mainly from 2020 onward, while a small number of foundational earlier works (for example, on design-science methodology) were retained. Sources were included when they addressed at least one framework dimension and were relevant to public-interest institutions, and were excluded when they lacked conceptual or methodological relevance, were promotional or non-retrievable, or duplicated already-included work. Screening proceeded from title and abstract to full-text assessment against these criteria. Table 1 summarizes the search strategy and selection criteria.

**Table 1:** Search strategy and selection criteria.

Element	Specification
Review type	Integrative review (Snyder, 2019) combined with design-science research (Hevner et al., 2004; Peffers et al., 2007)
Databases and sources	Scopus, Web of Science, IEEE Xplore, ACM Digital Library, SpringerLink; policy/standards sources (EUR-Lex, OECD.AI, UNESCO, NIST); preprint servers (arXiv, SSRN) for emerging technical work
Time window	Primarily 2020–2026, with selected foundational earlier works retained
Keyword clusters	(i) AI-generated/synthetic content/AIGC; (ii) provenance, content credentials, watermarking, fingerprinting; (iii) labeling, disclosure, transparency; (iv) privacy, security, data protection, generative AI; (v) AI governance, responsible AI, compliance, accountability
Inclusion criteria	Peer-reviewed studies, technical surveys, recognized governance frameworks, standards documents, and legal-policy analyses addressing at least one framework dimension and relevant to public-interest institutions
Exclusion criteria	Sources without conceptual or methodological relevance to governance; promotional or non-retrievable materials; duplicates
Screening	Title and abstract screening followed by full-text assessment against the inclusion criteria
Synthesis and analysis	Thematic mapping into four domains (provenance/authenticity; labeling/disclosure; privacy/safety; accountability/compliance); translation of recurring risks into architectural requirements and institutional controls
Design output	A layered, regulation-aware governance framework with a technical control stack, threat model, risk-tiered workflow, and evaluation metrics

The methodological process was organized into five stages. First, the study delimited the governance problem by identifying recurring institutional risks associated with AI-generated content, including unverifiable origin, weak disclosure, privacy exposure, harmful synthetic outputs, and insufficient accountability. Second, the literature was mapped across four thematic domains: content provenance and authenticity, labeling and disclosure, privacy and safety, and AI accountability and compliance. Third, findings from these domains were synthesized into a layered governance architecture, connecting technical safeguards with institutional responsibilities. Fourth, the architecture was operationalized through a technical control stack, a threat model, risk-tiered workflow checkpoints, and governance metrics. Fifth, the resulting framework was assessed for internal coherence by examining whether each layer addressed a distinct governance function while remaining interoperable with the other layers, and its applicability was examined through an illustrative analytical application to documented incidents (Section 5.9).

The analytic procedure consisted of translating literature-derived risks into architectural requirements and then translating those requirements into institutional controls. For example, the risk of unverifiable synthetic origin was converted into a provenance and authenticity layer supported by content credentials, cryptographic signatures, signed manifests, and metadata chains. The risk of misleading synthetic status was converted into a labeling and disclosure layer supported by visible labels, machine-readable tags, and tiered disclosure rules. Privacy and safety risks were translated into controls such as data minimization, access restrictions, restricted generation domains, content filtering, review gates, and escalation pathways. Finally, accountability risks were translated into audit logs, authorization trails, human validation checkpoints, incident reporting procedures, and retention policies. Following this design-science logic, the framework was required to be lifecycle-oriented, regulation-aware, risk-tiered, and evaluable through concrete indicators.

The method has limitations. Because the literature corpus was selected purposively rather than exhaustively, the synthesis may not capture every relevant contribution, and the integrative approach favors conceptual breadth over the systematic comparability of a PRISMA-style review. More fundamentally, because the study develops a conceptual architecture, it does not provide empirical validation through institutional pilots, controlled experiments, or benchmarking across deployed systems; the illustrative application in Section 5.9 supports analytic plausibility but not deployment effectiveness. The contribution is therefore architectural and normative-operational rather than experimental. This limitation is consistent with the purpose of the paper: to establish a structured governance model that can later be tested, adapted, and evaluated in educational institutions, public administrations, and other public-interest environments. Because the framework is conceptual and architecture-oriented, its value does not depend on a single institutional case, jurisdiction, or empirical setting; it identifies governance functions—provenance, labeling, privacy and safety safeguards, accountability trails, and review checkpoints—that institutions can adapt to their own contexts. This conceptual generality is treated here as a strength rather than a limitation.

## 4 The institutional compliance problem of AI-generated content

As AI-generated content becomes embedded in institutional workflows, the central challenge is no longer only whether synthetic content can be produced at scale, but whether its production, circulation, and use can remain compliant with legal, organizational, and procedural requirements. In universities, public agencies, and other public-interest institutions, generated content may affect assessment, administrative communication, records, guidance, and policy implementation (Capella, 2025). This means that AI-generated content is not merely a media authenticity issue; it is also a compliance issue involving provenance, disclosure, privacy, accountability, and traceability across organizational processes (El Ali et al., 2024). Recent surveys and governance studies show that these risks are now treated as systemic rather than incidental, especially where content moves from experimentation into formal institutional use.

The compliance problem emerges because the main control mechanisms currently discussed in the literature operate unevenly and often in isolation. Watermarking and fingerprinting research has advanced significantly, but the same literature also acknowledges persistent limitations related to robustness, spoofing, paraphrasing, transferability, and real-world interoperability (Liu et al., 2025). At the same time, provenance-oriented work argues that authenticity cannot be reduced to detection alone; institutions also need persistent metadata chains and verifiable histories of generation and transformation if they are to support auditability and downstream responsibility (Balan et al., 2025). In practical terms, this means that a piece of AI-generated content may be technically identifiable yet still fail organizational compliance requirements if its origin, modification history, or authorization pathway cannot be reconstructed.

A further layer of the problem is privacy and regulatory exposure. Generative systems can process, reproduce, or infer sensitive information in ways that complicate existing data governance models, especially when institutions rely on cloud services, external APIs, or weakly governed content pipelines (Patel et al., 2024). Recent privacy scholarship on generative AI shows that large-scale content generation amplifies risks of data leakage, improper reuse, and weak enforceability of user-centered protection models (Liu et al., 2024). Legal analyses of generative AI and copyright similarly indicate that disclosure, labeling, and provenance are becoming increasingly central to regulation, particularly where content authenticity and rights management intersect (Yu, 2025). For institutions, this creates a difficult compliance landscape: content that appears operationally useful may still create privacy, disclosure, or copyright vulnerabilities if governance is not built into the content lifecycle itself.

From an institutional perspective, the compliance problem can therefore be defined as the gap between technically generable content and organizationally governable content. A university or public institution may be able to generate, summarize, adapt, and distribute AI-produced content efficiently, yet still lack the mechanisms required to prove where the content came from, whether it was modified, what data risks it carries, whether labeling was appropriate, and who remains responsible for its use (Maryala, 2025). The growing literature on higher education AI policies and LLM lifecycle bias reinforces this point by showing that governance failures often arise not from one catastrophic error, but from the accumulation of uncoordinated design, deployment, and monitoring choices (Ahmad et al., 2026). For that reason, the next section proposes a regulation-aware framework that integrates provenance, privacy, safety, and accountability as mutually dependent design requirements for AI-generated content in education and public administration.

Illustrative cases make the institutional compliance problem concrete. In Mexico, media reports described a case involving a student from a public higher education institution who allegedly used artificial intelligence to manipulate photographs of female classmates into sexually explicit synthetic images, which were then circulated and commercialized (Milenio, 2023). Subsequent reporting indicated that the case advanced through criminal proceedings and resulted in a prison sentence in 2025. Even when anonymized for scholarly discussion, this case reveals that AI-generated content is no longer confined to abstract debates on misinformation or copyright, but already affects privacy, dignity, institutional duty of care, and the credibility of university governance.

Comparable international cases reinforce the same point. In the United States, the Associated Press reported in 2026 that two teenagers at a private school were sanctioned after using AI to create hundreds of fake nude images of dozens of girls using photographs gathered from social media and school-related sources (Scolforo, 2026). The case was publicly described in terms of severe emotional harm, institutional disruption, and the insufficiency of existing safeguards. Taken together, these incidents show that the compliance problem of AI-generated content is not limited to platform moderation or individual misuse; it is also an institutional governance problem requiring provenance, labeling, accountability, privacy protection, and response protocols. These two incidents are revisited in Section 5.9 as worked examples of how the proposed framework would apply.

## 5 A regulation-aware framework for governing AI-generated content

To respond to the institutional compliance problem identified in the previous section, this paper proposes a regulation-aware governance framework for AI-generated content. The framework is based on the premise that content governance cannot rely on a single safeguard, such as detection or disclosure, but must instead operate as an integrated architecture linking provenance, labeling, privacy protection, safety controls, and accountability (Singh, 2024). Recent research on text watermarking, provenance-aware authenticity, and responsible AI governance shows that isolated technical solutions are often fragile when confronted with paraphrasing, multimodal transformation, adversarial behavior, or weak institutional enforcement (Nemecek et al., 2025). For that reason, the framework proposed here treats AI-generated content governance as a layered institutional system rather than as a stand-alone technical fix.

### 5.1 Framework logic

The framework is organized around four interdependent layers. The first is the provenance and authenticity layer, which captures how AI-generated content is linked to its source, generation pathway, and transformation history (Spoczynski et al., 2025). The second is the labeling and disclosure layer, which determines how the synthetic character of content is communicated to users and institutions (Gamage et al., 2025). The third is the privacy and safety layer, which addresses risks associated with sensitive data exposure, impersonation, harmful manipulation, and downstream misuse (Golda et al., 2024). The fourth is the accountability and compliance layer, which connects all prior layers to organizational responsibilities, auditability, and regulatory obligations (Bignami et al., 2025). This layered structure is consistent with current findings showing that authenticity, user trust, privacy, and governance are mutually dependent rather than separable design problems.

Under this logic, AI-generated content should not be governed only after publication or circulation. Instead, governance must be embedded throughout the content life cycle, from generation and transformation to storage, dissemination, institutional use, and post hoc review (Jayaram et al., 2024). This is especially relevant in higher education and public administration, where synthetic content may be inserted into workflows such as policy interpretation, student communication, guidance systems, public information releases, or complaint handling (Jiang et al., 2025). In these environments, governance failures are not merely reputational; they can produce procedural harm, privacy violations, and organizational liability (Chen and Hu, 2025). The framework therefore shifts the focus from “Can the content be detected?” to “Can the content be institutionally governed across its life cycle?”

### 5.2 Core layers of the framework

The provenance and authenticity layer requires that AI-generated content be associated with verifiable information about origin, authorship conditions, generation tools, and relevant transformations. This layer does not assume that provenance alone solves the problem, but it treats provenance as the minimum condition for any later claim of authenticity, responsibility, or compliance (Longpre et al., 2024). Recent work on content authenticity in the age of AI emphasizes that provenance has value not only for detection, but for institutional audit, dispute resolution, and trust calibration in environments where content may be copied, edited, or recontextualized (Feng et al., 2023). In practice, this layer may involve cryptographic signatures, content credentials, metadata chains, or persistent attribution records depending on the technical environment.

The labeling and disclosure layer addresses the communication problem that follows from provenance. Even if synthetic origin can be technically documented, institutions still need rules for when, how, and to whom such information should be disclosed. Recent experimental work shows that AI-content labels can reduce perceived believability in some contexts, but their effects are not uniform and may depend on wording, placement, and user expectations (Wittenberg et al., 2025). This implies that labeling should not be treated as a purely symbolic compliance gesture, but as a governance instrument that must be calibrated to use case, audience, and risk level (d'Angelo et al., 2022). For educational and public-sector institutions, the framework therefore recommends tiered disclosure rules: stronger disclosure for public-facing or decision-relevant content, and context-sensitive disclosure for internal or low-stakes use.

The privacy and safety layer focuses on the harms that may arise even when provenance or labeling exists. AI-generated content can still expose personal information, simulate identities, amplify bias, or facilitate abuse if institutions lack safeguards on data inputs, access controls, or content reuse (Al Breiki and Mahmoud, 2025). Recent research on privacy and responsible AI governance shows that safety and privacy cannot be treated as downstream corrections; they must be embedded into design choices regarding data minimization, restricted generation domains, secure storage, content review, and escalation pathways for harmful synthetic outputs (Wang et al., 2025). In educational and administrative settings, this layer is especially important where generated content may concern students, staff, or public-facing information.

The accountability and compliance layer integrates the previous layers into institutional decision-making. Its purpose is to ensure that generated content remains subject to assignable responsibility, auditability, and review under relevant legal or organizational rules (Oduro et al., 2022). This layer requires institutions to define who authorizes content generation, who validates or edits outputs, which logs are retained, how incidents are escalated, and how compliance is demonstrated under privacy, copyright, platform, or sectoral obligations (Ramsaran, 2025). In this sense, the framework is regulation-aware because it assumes that governance mechanisms must be designed not only for technical robustness but also for regulatory defensibility (Imperial et al., 2025).

### 5.3 Technical control stack for regulation-aware governance

To translate the proposed framework into a governable institutional architecture, each layer must be supported by a corresponding technical control stack. The provenance and authenticity layer can be operationalized through content credentials, cryptographic signatures, signed manifests, persistent metadata chains, and transformation logs that preserve verifiable links between generated content and its origin (Simmons and Winograd, 2024; Spoczynski et al., 2025). These controls are especially important in institutional settings because provenance cannot depend on downstream detection alone; it must remain auditable even after storage, dissemination, or internal reuse (Longpre et al., 2024).

The labeling and disclosure layer requires controls that make synthetic status both visible and machine-readable. In practice, this may involve user-facing labels, metadata-based disclosure tags, tiered warning messages, and policy-driven rules specifying when synthetic origin must be disclosed more explicitly, particularly for public-facing or decision-relevant content (Wittenberg et al., 2025; Gamage et al., 2025). This layer should therefore be treated not as a symbolic transparency mechanism, but as a structured disclosure system linked to use case, audience, and institutional risk.

The privacy and safety layer depends on controls that restrict harmful generation, limit sensitive-data exposure, and reduce misuse opportunities across the content life cycle. Relevant mechanisms include data minimization, secure storage, role-based access control, content filtering, restricted generation domains, review gates, and escalation pathways for harmful or rights-infringing outputs (Al Breiki and Mahmoud, 2025; Wang et al., 2025). In universities and public administrations, these safeguards are especially relevant where generated content may concern students, staff, records, or public communication.

The accountability and compliance layer requires institutional controls that connect technical events with assignable human and organizational responsibility. These controls include authorization trails, audit logs, human validation checkpoints, incident reporting channels, retention policies, and review thresholds for high-stakes use cases (Oduro et al., 2022; Eisenberg et al., 2025). Under this logic, governance becomes regulation-aware not only because institutions adopt disclosure or provenance mechanisms, but because those mechanisms are embedded into an auditable architecture capable of supporting privacy, safety, traceability, and legal defensibility at the same time. Table 2 summarizes the technical control stack across the four layers.

**Table 2:** Technical control stack for regulation-aware governance of AI-generated content.

Layer	Main institutional risk	Representative technical controls	Governance function
Provenance and authenticity	Unknown origin, unverifiable transformation history	Content credentials, cryptographic signatures, signed manifests, metadata chains, provenance logs	Traceability and authenticity verification
Labeling and disclosure	Misleading synthetic status, inadequate disclosure	Visible labels, machine-readable tags, tiered disclosure rules, policy-driven warning messages	Transparency and audience-calibrated communication
Privacy and safety	Sensitive-data exposure, impersonation, harmful synthetic outputs	Data minimization, access controls, secure storage, restricted generation domains, content filtering, and review gates	Risk reduction and rights protection
Accountability and compliance	Unassignable responsibility, weak auditability	Authorization trails, audit logs, human validation checkpoints, incident reporting, retention policies	Regulatory defensibility and institutional oversight

Table 2 shows that regulation-aware governance depends on the coordination of multiple technical and organizational controls rather than on any single mechanism. In particular, provenance, labeling, privacy and safety, and accountability operate as mutually reinforcing layers. This supports the paper's central argument that AI-generated content must be governed as an institutional architecture rather than as an isolated detection or disclosure problem.

### 5.4 Threat model and failure modes

A regulation-aware governance architecture must also specify the main failure modes and adversarial conditions that can undermine institutional control over AI-generated content. In practice, governance does not fail only when content is falsely accepted as authentic, but also when provenance is stripped, labels are removed or ignored, metadata chains are broken, sensitive information is exposed, or synthetic outputs are reused in harmful or misleading contexts. For this reason, the proposed framework is complemented by a threat-oriented perspective that links each governance layer to specific risks of failure or circumvention.

At the provenance and authenticity level, a central threat is metadata loss or provenance stripping during content transfer, conversion, reposting, or institutional reuse. In such cases, content may remain technically generated yet become operationally detached from its source, transformation history, or authorization pathway. A second threat concerns paraphrasing, editing, or transformation attacks that weaken watermark robustness and reduce the reliability of origin signals. These risks are especially relevant where content moves across platforms or between institutional and public-facing environments.

At the labeling and disclosure level, a major failure mode is ineffective or inconsistent signaling. Labels may be absent, poorly worded, visually weak, removed in downstream circulation, or ignored by users under conditions of high trust or urgency. This means that disclosure cannot be treated as a purely formal obligation; it must be resilient enough to remain meaningful under realistic communication conditions. In high-stakes institutional settings, misleadingly unlabeled or weakly labeled synthetic content may create procedural harm even when provenance information exists somewhere in the background.

At the privacy and safety level, the main threats include sensitive-data leakage, identity simulation, harmful synthetic manipulation, and secondary misuse of generated content. These harms may arise through insecure data pipelines, untrusted external APIs, poorly constrained generation domains, or weak review and escalation procedures. Particularly serious cases include non-consensual intimate imagery, impersonation, defamatory content, manipulative public-facing communication, or outputs that expose personal or institutional information. From a governance perspective, these are not merely misuse events, but failures of privacy-preserving and safety-aware design.

At the accountability and compliance level, failure occurs when content-related events cannot be reconstructed in a way that supports assignable responsibility, auditability, or incident response. Missing logs, unclear validation roles, absent authorization records, and weak retention policies can all make it impossible to determine who generated, edited, approved, or disseminated synthetic content. Under such conditions, technical controls may exist in isolation, yet institutional compliance still fails because the organization cannot demonstrate traceability, defensibility, or procedural control.

Taken together, these failure modes show that AI-generated content governance must be designed not only for nominal operation, but also for degradation, adversarial manipulation, and institutional misuse scenarios. For this reason, the framework proposed here treats threat modeling as an integral part of regulation-aware governance rather than as a separate downstream security exercise. Table 3 summarizes the main failure modes and corresponding control responses.

**Table 3:** Main failure modes and control responses in regulation-aware governance of AI-generated content.

Failure mode	Illustrative risk	Affected layer	Representative control response
Provenance stripping	Loss of source and transformation history during transfer or reuse	Provenance and authenticity	Cryptographic signatures, signed manifests, persistent metadata chains, and provenance logs
Paraphrasing or transformation attack	Weakening of watermark or origin signals through editing or reformatting	Provenance and labeling	Combined provenance verification, watermark robustness checks, and secondary disclosure controls
Weak or absent labeling	Synthetic content circulates without meaningful disclosure	Labeling and disclosure	Tiered labeling rules, machine-readable tags, and policy-driven disclosure thresholds
Sensitive-data leakage	Generated content reveals personal or institutional information	Privacy and safety	Data minimization, access controls, secure storage, restricted generation domains
Identity simulation or harmful manipulation	Non-consensual imagery, impersonation, deceptive public-facing content	Privacy and safety	Content filtering, review gates, escalation pathways, and incident response protocols
Missing accountability trail	Inability to reconstruct who generated, approved, or disseminated content	Accountability and compliance	Audit logs, authorization records, human validation checkpoints, and retention policies

Table 3 makes explicit that governance failures can occur even when some controls are present, for example when provenance is stripped, labels are ineffective, or accountability trails are incomplete. This reinforces the need for a threat-aware approach in which technical safeguards are evaluated not only under nominal operation, but also under adversarial or degraded conditions. In this sense, the framework is designed to address both routine governance and institutional failure scenarios.

### 5.5 Operational workflow and governance checkpoints

Operationally, the technical controls and failure scenarios described above can be integrated into a five-stage governance workflow. The purpose of this workflow is to ensure that AI-generated content is not governed only after publication or misuse, but throughout its institutional life cycle. In public-interest environments such as universities and public administrations, governance must therefore begin before content is generated and continue through disclosure, storage, dissemination, oversight, and incident response.

The first stage is use-case classification. At this stage, institutions determine whether the intended use of AI-generated content is low-stakes, medium-stakes, or high-stakes according to audience, decision relevance, privacy sensitivity, and potential harm. Low-stakes uses may include internal drafting or exploratory content support. Medium-stakes uses may involve institutional communication, educational guidance, or public-facing informational content (Watkins et al., 2021). High-stakes uses include content embedded in decision-support, administrative interpretation, student-related records, complaint handling, or any workflow with legal, procedural, or rights-related implications. This classification step is essential because governance intensity should be proportional to institutional risk rather than uniformly applied across all generated content.

The second stage is provenance and disclosure activation. Once the use case has been classified, the institution activates the corresponding provenance, authenticity, and labeling controls. In low-stakes settings, lightweight provenance and internal disclosure may be sufficient (Agarwal and Nene, 2025). In medium-stakes settings, institutions should require stronger metadata persistence and visible synthetic-content labeling. In high-stakes settings, provenance records, disclosure obligations, authorization checks, and human review should all be activated as mandatory safeguards. In this way, provenance and labeling become dynamic governance instruments calibrated to risk level rather than static compliance gestures.

The third stage is privacy and safety review. Before dissemination or institutional use, the content must be assessed for sensitive-data exposure, impersonation risk, harmful manipulation, rights-infringing content, or unsafe downstream use (Tabassi, 2023). This review may involve automated screening, access restrictions, domain constraints, and human escalation depending on the risk tier. The key principle is that content should not move into institutional circulation if privacy and safety conditions remain unresolved, even when provenance or labeling mechanisms are already in place.

The fourth stage is accountability validation. At this point, the institution verifies whether the content has an assignable authorization trail, whether the appropriate reviewer or responsible unit has validated it, and whether the relevant logs and records are retained (Jain et al., 2024). This stage is what transforms a technically controlled system into an institutionally governable one. Without accountability checkpoints, even well-labeled or provenance-linked synthetic content may still fail compliance because responsibility cannot be reconstructed after the fact.

The fifth stage is post-deployment monitoring and incident response. Once content has entered circulation, institutions must retain the capacity to audit provenance, review logs, investigate incidents, issue corrections, and activate escalation or takedown procedures when harm occurs. This stage is especially important in the case of manipulated, recontextualized, or maliciously reused synthetic content (Taeihagh, 2025). Under the proposed framework, governance is therefore both preventive and responsive: preventive in activating safeguards before content use, and responsive in preserving institutional capacity to investigate and respond after deployment.

Taken together, this workflow shows that regulation-aware governance of AI-generated content is not a one-time filter, but a recurring process of classification, control activation, review, validation, and monitoring. Its practical value lies in translating abstract principles such as transparency, privacy, and accountability into concrete governance checkpoints that can be embedded into institutional workflows. Table 4 summarizes how the workflow can be calibrated according to institutional risk level.

**Table 4:** Risk-tiered operational workflow for governing AI-generated content.

Workflow stage	Low-stakes use	Medium-stakes use	High-stakes use
1. Use-case classification	Internal drafting or exploratory support	Institutional communication, educational guidance	Decision-support, records, complaint handling, rights-sensitive workflows
2. Provenance and disclosure activation	Basic provenance and internal disclosure	Visible labeling and stronger metadata persistence	Mandatory provenance, disclosure, authorization, and review
3. Privacy and safety review	Basic screening	Automated and procedural review	Human escalation, domain restrictions, strict safeguards
4. Accountability validation	Minimal logging	Assigned reviewer and retained records	Formal authorization trail, audit logs, validation checkpoints
5. Monitoring and incident response	Basic correction capacity	Log review and response procedures	Auditability, takedown, escalation, legal and institutional response

Table 4 shows that governance intensity should vary according to institutional risk level rather than be applied uniformly across all AI-generated content. Low-stakes uses may tolerate lighter controls, whereas high-stakes contexts require stronger provenance, review, and accountability mechanisms. This tiered logic is what allows the framework to remain both operationally feasible and regulation-aware. Under this model, the governance of AI-generated content becomes an operational architecture rather than a *post-hoc* compliance exercise, which is precisely what institutions require when synthetic content affects public trust, procedural integrity, and legal defensibility.

### 5.6 Evaluation criteria and governance metrics

A regulation-aware governance architecture also requires evaluation criteria capable of translating institutional expectations into observable controls and measurable outcomes. If AI-generated content is to be governed as an infrastructure of institutional risk and responsibility, then provenance, labeling, privacy, safety, and accountability cannot remain only conceptual layers; they must also be assessable through implementation-oriented metrics (Golda et al., 2024). This is especially important in public-interest environments, where governance effectiveness depends not only on whether controls exist in principle, but on whether institutions can demonstrate that those controls function in practice.

At the provenance and authenticity level, evaluation should focus on whether generated content retains verifiable source and transformation information across storage, transfer, and reuse. Relevant criteria include the proportion of synthetic-content items with preserved provenance metadata, the proportion of outputs linked to signed manifests or verifiable credentials, and the percentage of content objects whose generation pathway can be reconstructed during audit (Novelli et al., 2024). At the labeling and disclosure level, evaluation should focus on the consistency, visibility, and contextual appropriateness of disclosure, including the proportion of public-facing synthetic content correctly labeled and the rate at which labels remain preserved after internal redistribution or platform transfer (Singh, 2024).

At the privacy and safety level, governance metrics should capture both prevention and incident response, including the number of sensitive-data exposure events, the proportion of restricted-domain requests blocked or escalated, the number of harmful synthetic outputs detected before dissemination, and the average response time for safety-related incidents (Leslie et al., 2023). At the accountability and compliance level, evaluation should examine whether synthetic content remains linked to assignable responsibility and reviewable records, including the proportion of outputs with complete authorization trails, the percentage of high-stakes outputs validated by a designated reviewer, the completeness of retained logs, and the average time required to reconstruct an accountability chain during audit (Feng et al., 2023).

In response to the need for greater practical applicability, Table 5 extends these indicators with an explicit account of how each metric can be measured and which institutional records or systems supply the underlying data. This is intended to help universities and public administrations move from abstract indicators to concrete measurement procedures embedded in existing information systems, audit processes, and incident-management workflows (Ramsaran, 2025).

**Table 5:** Evaluation criteria and indicative measurement approaches for regulation-aware governance.

Governance dimension	Illustrative evaluation metric	Indicative measurement approach and data source	Governance purpose
Provenance and authenticity	Percentage of content items with preserved provenance metadata	Automated audit of metadata fields in the content-management or document system; ratio of items retaining manifests to total generated items	Traceability and auditability
Provenance and authenticity	Percentage of outputs linked to verifiable credentials or signed manifests	Verification logs from the signing/credentialing service; periodic sampling during internal audit	Authenticity verification
Labeling and disclosure	Percentage of public-facing synthetic content correctly labeled	Spot-check audit of published channels against a disclosure register; automated label-detection where available	Transparent disclosure
Labeling and disclosure	Label-retention rate after internal redistribution or platform transfer	Before/after comparison of labels on a sampled content set across redistribution points	Context-sensitive compliance
Privacy and safety	Number of sensitive-data exposure incidents	Incident-management and data-protection (DPO) records over a fixed period	Risk monitoring
Privacy and safety	Percentage of harmful or restricted outputs blocked before dissemination	Logs from content filters and review gates; ratio of blocked/escalated to flagged requests	Preventive safety control
Privacy and safety	Average response time to privacy or safety incidents	Timestamps from incident-response tickets (detection to resolution)	Responsive governance capacity
Accountability and compliance	Percentage of outputs with a complete authorization trail	Audit of authorization and workflow logs against a defined completeness checklist	Assignable responsibility
Accountability and compliance	Percentage of high-stakes outputs validated by a designated reviewer	Review-system records cross-referenced with use-case classification	Human oversight
Accountability and compliance	Time required to reconstruct an accountability chain during audit	Timed reconstruction exercise during internal or external audit	Regulatory defensibility

Table 5 shows that the proposed framework can be assessed through concrete institutional indicators, each tied to a measurement procedure and a data source, rather than only through abstract principles. This is important because governance becomes operationally meaningful only when institutions can verify whether provenance is retained, labeling rules are followed, privacy and safety safeguards are functioning, and accountability remains auditable. In that sense, the framework is designed not only for conceptual guidance, but also for future implementation assessment and benchmarking.

### 5.7 Positioning relative to existing AI governance frameworks

A recurring question for any governance proposal is how it advances or differs from established models. The framework proposed here does not seek to replace existing AI-governance instruments; it specializes and operationalizes them for AI-generated content in public-interest institutions. Several instruments inform AI governance broadly. The NIST AI Risk Management Framework offers a voluntary, system-level structure organized around the Govern, Map, Measure, and Manage functions (Tabassi, 2023). The OECD AI Principles articulate values-based commitments to transparency, accountability, robustness, and human-centered AI (OECD, 2019). The EU AI Act establishes a risk-based legal regime that, among other duties, requires disclosure of certain AI-generated or manipulated content (European Parliament Council of the European Union, 2024). The UNESCO Recommendation provides a global, human-rights-based ethics standard with policy action areas that explicitly include education (UNESCO, 2021). The GDPR constrains the processing of personal data (European Parliament and Council of the European Union, 2016), and ISO/IEC 42001 specifies a management-system approach to AI (International Organization for Standardization, 2023). Complementary scholarship proposes enterprise-wide unified control frameworks (Eisenberg et al., 2025) and multi-layer governance models that integrate regulation, standards, and certification (Agarwal and Nene, 2025).

These instruments share two features that the proposed framework is designed to address. First, they are largely system- or organization-centric and treat AI-generated content as one risk among many rather than as a distinct life-cycle object with its own provenance, labeling, privacy, and accountability requirements. Second, they articulate principles and obligations but offer limited guidance on how these dimensions should be combined operationally across the content life cycle in education and public administration. Table 6 positions the framework against these instruments.

**Table 6:** Positioning of the proposed framework relative to existing AI governance instruments.

Instrument/framework	Primary orientation	Relation to and gap addressed by the proposed framework
NIST AI RMF (Tabassi, 2023)	Voluntary, system-level AI risk management (Govern–Map–Measure–Manage)	General and system-centric; the framework specializes this logic to the AI-generated content life cycle and adds provenance and labeling controls
OECD AI Principles (OECD, 2019)	Values-based intergovernmental principles	High-level; the framework operationalizes transparency, accountability, and robustness as institutional content controls and metrics
EU AI Act (European Parliament Council of the European Union, 2024)	Risk-based hard law with disclosure duties for synthetic/manipulated content	Defines obligations but not institutional implementation; the framework maps duties to deployable controls and workflows
UNESCO (2021)	Global, human-rights-based ethics standard	Normative; the framework provides an architecture to translate ethics into content governance in education and public administration
GDPR (European Parliament and Council of the European Union, 2016)	Data-protection law	Constrains data handling; the framework embeds minimization, purpose limitation, and security in its privacy-and-safety layer
ISO/IEC 42001 (International Organization for Standardization, 2023)	AI management-system standard (Plan–Do–Check–Act)	Process/management focus; the framework adds content-specific, life-cycle controls and evaluation indicators
Unified/layered governance models (Eisenberg et al., 2025; Agarwal and Nene, 2025)	Enterprise-wide integration of AI governance	Broad and system-oriented; the framework is narrower and deeper, focused on synthetic content as an institutional risk object across its life cycle

The distinctive contribution of the framework is therefore not the invention of new principles but the integration of provenance, labeling, privacy and safety, and accountability into a single, life-cycle-oriented architecture, complemented by a threat model, a risk-tiered workflow, and evaluation metrics tailored to education and public administration. Where existing instruments specify what institutions must achieve, the framework specifies how the corresponding controls can be combined and operated as a coherent system.

### 5.8 Regulatory grounding of the framework

Because the framework is regulation-aware, its layers are designed to align with concrete legal and standards-based obligations rather than with abstract principles alone. The EU AI Act introduces transparency duties requiring that certain AI-generated or manipulated content be disclosed to users, alongside record-keeping, risk-management, data-governance, human-oversight, and post-market-monitoring obligations for higher-risk uses (European Parliament Council of the European Union, 2024; Novelli et al., 2024). The GDPR constrains the processing of personal data within generative pipelines through principles of data minimization and purpose limitation, requirements for data protection by design and by default, and obligations regarding the security of processing (European Parliament and Council of the European Union, 2016). Soft-law and standards instruments reinforce these expectations: the OECD AI Principles and the UNESCO Recommendation emphasize transparency, accountability, and human oversight OECD, 2019; UNESCO, 2021), while the NIST AI RMF and ISO/IEC 42001 provide risk-management and management-system structures within which such controls can be embedded (Tabassi, 2023; International Organization for Standardization, 2023). Table 7 provides an indicative mapping of framework layers to these obligations.

**Table 7:** Indicative mapping of framework layers to regulatory and standards-based obligations.

Framework layer	Illustrative obligations and provisions	Source instruments (indicative)
Provenance and authenticity	Record-keeping and traceability; documentation of generation and transformation	EU AI Act Arts. 12, 50; NIST AI RMF (Map/Measure); OECD accountability
Labeling and disclosure	Transparency duties; disclosure that content is AI-generated or manipulated	EU AI Act Art. 50; OECD transparency and explainability; UNESCO transparency
Privacy and safety	Data minimization, purpose limitation, data protection by design, security of processing; risk management	GDPR Arts. 5, 25, 32; EU AI Act Arts. 9–10; UNESCO data-governance action area
Accountability and compliance	Accountability, human oversight, documentation, and post-market monitoring	EU AI Act Arts. 14, 17, 72; GDPR Art. 5(2); NIST AI RMF (Govern); ISO/IEC 42001

The mappings in Table 7 are indicative rather than prescriptive: they do not constitute legal advice, and the specific obligations that apply depend on the jurisdiction and on whether an institution acts as a provider or deployer of a given system. Nonetheless, the mapping demonstrates that the framework's layers are not arbitrary but correspond to obligations that institutions already face or will soon face. Conversely, it shows that satisfying these instruments in practice requires precisely the kind of operational controls the framework specifies, which is the core sense in which the proposed architecture is regulation-aware.

### 5.9 Illustrative application of the framework

Although the framework is conceptual, its applicability can be examined through an illustrative analytical application to documented incidents, a recognized form of preliminary demonstration for design-oriented artifacts (Peffers et al., 2007). The two incidents introduced in Section 4—the case at a Mexican higher education institution (Milenio, 2023) and the case at a United States school (Scolforo, 2026)—are used here as anonymized worked examples. In both, an individual used generative tools to produce non-consensual sexual imagery of identifiable students from ordinary photographs, after which the content circulated and, in one case, was commercialized. The point of the exercise is not to assign blame but to show how the framework's layers would structure institutional prevention, detection, and response.

Examined through the framework, the harm in both cases did not stem from a single technical failure but from the absence of an integrated governance architecture. At the provenance and authenticity level, the manipulated images circulated without verifiable origin, and institutions held no records linking the generation tools or actors to the outputs. At the labeling and disclosure level, manipulated content was presented as authentic, with no mechanism to signal its synthetic character. At the privacy and safety level, there were weak safeguards against the production and dissemination of non-consensual intimate imagery involving identifiable students, and no escalation or victim-protection pathway designed for this harm. At the accountability and compliance level, institutions had limited capacity to detect, attribute, and respond before the matter escalated externally or into criminal proceedings. Table 8 summarizes how the framework would map onto these governance gaps.

**Table 8:** Illustrative application of the framework to documented incidents of non-consensual synthetic imagery.

Framework layer	Governance gap revealed by the cases	Control the framework would activate
Provenance and authenticity	Synthetic images circulated without verifiable origin; no institutional record of generation	Content credentials and manifests; provenance logging within institutional systems
Labeling and disclosure	Manipulated images presented as authentic, with no disclosure	Mandatory disclosure and labeling for manipulated imagery; detection-triggered flags
Privacy and safety	Non-consensual intimate imagery of identifiable students; severe dignity and privacy harm	Restricted generation domains, content filtering, escalation and victim-protection protocols
Accountability and compliance	Limited capacity to detect, attribute, and respond before external or legal escalation	Authorization trails, incident-response protocol, designated responsible unit, retention and reporting

Applying the workflow of Section 5.5, use-case classification would place such content in the high-stakes, rights-sensitive tier, triggering mandatory provenance and disclosure controls, strict privacy-and-safety review with escalation pathways, and a formal accountability trail with incident response. This illustrative application does not constitute empirical validation: it does not measure deployment effectiveness, institutional cost, or comparative performance, and the institutions involved did not implement the framework. Its purpose is to demonstrate the framework's analytic traction and to specify the controls whose absence is associated with governance failure, thereby motivating the empirical studies proposed in Section 7. Read together with the comparative positioning (Section 5.7) and the regulatory mapping (Section 5.8), it shows that the framework is both differentiated from existing models and connected to the concrete settings in which AI-generated content already causes institutional harm. The cases therefore support the plausibility and relevance of the framework—showing how it can structure the analysis of real institutional risks to privacy, dignity, traceability, and the institutional duty of care—but they do not establish its implementation effectiveness.

## 6 Discussion and policy implications

The framework proposed in this paper suggests that the governance of AI-generated content should be understood as an institutional systems problem rather than as a narrow issue of detection or disclosure. This matters because much of the current debate still treats labeling, watermarking, provenance, privacy, and accountability as separate interventions, even though their practical effectiveness depends on how they interact across the full content life cycle (Ramsaran, 2025). The technical control stack summarized in Table 2 makes this interdependence explicit by showing that provenance, disclosure, privacy and safety safeguards, and accountability controls are mutually reinforcing rather than functionally isolated (Deng et al., 2024). From this perspective, the paper advances the literature by arguing that the governability of AI-generated content depends less on any single safeguard and more on the coherence of the institutional architecture that binds those safeguards together.

Read through a normative lens, the framework can be understood as an exercise in institutional design that allocates normative functions, not merely technical controls. Provenance mechanisms create the evidentiary conditions under which responsibility for a piece of content can later be established; labeling rules operate as disclosure norms that govern what must be revealed to affected parties; privacy and safety safeguards define limits on permissible institutional action rather than mere performance targets; and audit logs, authorization trails, and review checkpoints allocate answerability by fixing who must be able to give reasons for how content was generated, approved, and used (Bovens, 2007). Framing the artifact this way clarifies that its purpose is normative institutional design: it specifies the conditions under which the use of AI-generated content by a public-interest institution can be justified, contested, and reviewed.

### 6.1 Distinguishing responsibility, accountability, and related concepts

This normative reading also requires distinguishing several concepts that are often used interchangeably but are analytically distinct. Table 9 separates them and links each to the mechanism in the proposed framework that supports it, so that the framework's claims about governance can be stated precisely.

**Table 9:** Key governance concepts and the framework mechanisms that support them.

Concept	Core meaning	Supporting mechanism in the framework
Traceability	Whether the origin and transformation history of AI-generated content can be reconstructed.	Provenance and content-credential metadata; version and transformation logs.
Auditability	Whether records and procedures exist to support subsequent review.	Audit logs, retained metadata, and documented, repeatable governance procedures.
Accountability	Whether institutional actors can be required to justify how content was generated, approved, or used.	Accountability trail, role assignment, and review checkpoints across the life cycle.
Answerability	The duty to provide reasons for a decision within an institutional setting.	Human-review checkpoints and disclosure obligations that require a justification before use.
Responsibility	The allocation to a specific actor of the obligation or role for a task or outcome.	Authorization trails and role-based ownership at each life-cycle stage.
Liability	The possible legal consequences attaching to an act or omission.	Regulatory grounding and compliance mapping (e.g., AI Act, data-protection law) and incident response.

### 6.2 Transparency, privacy, and the proportionality constraint

Making these functions explicit also surfaces a normative tension internal to regulation-aware design: transparency and privacy can pull in opposite directions. Stronger provenance, longer metadata retention, and broader disclosure improve traceability and accountability, but the same measures can expose personal data, sensitive institutional information, or protected records—a particularly acute risk in education and public administration, where the content at issue often concerns identifiable students, staff, or citizens. Institutional legitimacy in these settings depends not only on transparency but also on proportionality, data minimization, procedural fairness, and respect for rights. The framework therefore treats disclosure and retention not as goods to be maximized but as controls to be calibrated: provenance and audit data should be collected and retained to the extent necessary for accountability and no further, access should be governed by role and purpose, and high-stakes, rights-sensitive content should trigger the stricter privacy-and-safety review already specified in the risk-tiered workflow. Recognizing this tension explicitly is part of what distinguishes an operational governance architecture from a purely transparency-maximizing one.

A first implication concerns institutional strategy, particularly in education and public administration. The literature reviewed and the framework developed here indicate that organizations should not adopt AI-generated content technologies solely on the basis of productivity gains or ease of deployment (Chen and Hu, 2025). Instead, they must evaluate whether their governance capacity is sufficient to support provenance tracking, disclosure rules, privacy-preserving workflows, and incident response (Yu, 2025). This is especially important in universities and public institutions, where content may influence assessment, communication, records, guidance, and public-facing decisions (Gamage et al., 2025). In such environments, the failure to distinguish between low-stakes synthetic content and high-stakes decision-relevant content may generate institutional risk even when the underlying model performs well.

A second implication is policy-oriented. The framework reinforces the idea that regulation of AI-generated content should move beyond broad transparency obligations and toward more operational requirements tied to provenance, traceability, disclosure, and assignable responsibility (Pinho et al., 2025; Zhao et al., 2025). Current legal and policy debates already point in this direction, particularly in relation to copyright, labeling duties, and AI governance standards, but the literature also shows that these obligations remain difficult to implement unless institutions have corresponding internal controls and technical infrastructures (Ahmad et al., 2026). This means that policy effectiveness depends on regulation-aware design: rules must be translatable into deployable system requirements such as metadata persistence, access controls, logging, review thresholds, and verification protocols (Longpre et al., 2024).

A third implication concerns public trust and organizational legitimacy. AI-generated content challenges trust not only because synthetic outputs may be misleading, but because institutions may fail to communicate clearly when, how, and under what controls such content is used. Recent studies on labeling and authenticity suggest that users do not respond uniformly to synthetic content disclosures, which implies that institutional trust cannot be secured by labels alone (Feng et al., 2023). Rather, trust depends on whether institutions can demonstrate that synthetic content is governed through reliable provenance, meaningful disclosure, privacy safeguards, and visible accountability procedures (Oduro et al., 2022). For education and public administration, where legitimacy depends heavily on procedural integrity and public confidence, this finding is particularly significant.

A fourth implication concerns implementation and institutional benchmarking. The evaluation criteria summarized in Table 5 show that the proposed framework can be assessed through observable governance metrics, each tied to a measurement procedure, rather than only through abstract principles (Watkins et al., 2021). This is important because a regulation-aware architecture becomes meaningful in practice only when institutions can verify whether provenance is retained, labeling rules are followed, privacy and safety safeguards are functioning, and accountability remains auditable (Leslie et al., 2023). In this sense, the paper moves beyond a conceptual recommendation and offers a basis for future implementation studies, comparative benchmarking, and governance-performance assessment across institutions.

Alongside these implications, a regulation-aware architecture faces real implementation challenges and tensions that temper its adoption. First, several of the controls it presupposes are technically immature: provenance and watermarking mechanisms remain vulnerable to stripping, paraphrasing, and transformation, and interoperable content-credential infrastructures are not yet widely deployed (Liu et al., 2025; Nemecek et al., 2025). Institutions may therefore be obliged to disclose and trace content using tools that are themselves fragile. Second, institutional constraints are uneven. Well-resourced universities and agencies may be able to staff review boards, maintain audit logs, and procure provenance tooling, whereas under-resourced institutions—often those serving the most vulnerable populations—may lack the expertise, budget, or legacy-system flexibility to do so. There is thus a risk that regulation-aware governance widens rather than narrows institutional inequalities unless implementation support is provided.

Third, there is a genuine tension between transparency and privacy. The provenance logging, audit trails, and accountability records that make synthetic content governable can themselves accumulate sensitive personal data and create surveillance risks, placing the accountability layer in friction with the data-minimization commitments of the privacy layer and of the GDPR (European Parliament and Council of the European Union, 2016). Disclosure can likewise interact with profiling and chilling effects if labeling reveals who produced or modified content. The framework does not dissolve this tension; it makes it explicit and assigns it to the privacy-and-safety review stage, where logging scope and retention must be deliberately bounded. Fourth, applying the framework across regulatory environments is difficult: obligations differ markedly across the European Union, the United States, and Latin American jurisdictions; instruments such as the EU AI Act carry extraterritorial effects; and a control that satisfies one regime may sit uneasily with another, so institutions operating across borders must reconcile divergent and sometimes incompatible requirements. These challenges do not undermine the framework, but they qualify it: regulation-aware governance is best understood as a structured way of making such trade-offs explicit and assignable, not as a means of eliminating them.

Finally, this discussion opens an agenda for future interdisciplinary work at the intersection of advanced technologies and regulation. Technical research still needs stronger benchmarks for provenance robustness, watermark persistence, and privacy-preserving generation under realistic adversarial conditions, while policy research needs clearer models for translating emerging obligations into enforceable institutional practices (Spoczynski et al., 2025). The framework proposed here provides a bridge between these domains by treating security, privacy, safety, and regulation as mutually dependent components of content governance (Liu et al., 2024). In that sense, the main policy implication of the paper is that AI-generated content should be governed as an infrastructure of institutional risk and responsibility, not merely as an output type requiring ex post moderation (d'Angelo et al., 2022).

## 7 Conclusions and future work

This paper has argued that the governance of AI-generated content should be understood as a problem of institutional compliance and technical architecture rather than merely as a challenge of detection or a symbolic issue of disclosure. The central claim is that synthetic content becomes organizationally consequential when it enters educational and public-administration workflows in which authenticity, privacy, accountability, safety, and legal defensibility matter. Under those conditions, isolated safeguards are insufficient. What is required is a regulation-aware architecture that links provenance, labeling, privacy and safety controls, accountability mechanisms, and operational review processes into a coherent governance system.

The main contribution of the paper is the proposal of a layered architecture that integrates provenance and authenticity, labeling and disclosure, privacy and safety safeguards, accountability and compliance controls, and a risk-tiered operational workflow. By connecting these elements—and by positioning them against established instruments and mapping them to concrete regulatory obligations—the paper moves beyond fragmented approaches that treat watermarking, labels, privacy protections, or audit obligations as independent fixes. Instead, it positions AI-generated content governance as a life-cycle problem that must be addressed from generation to institutional use, downstream circulation, and post hoc review, with a scope deliberately delimited to the institutional governance of synthetic content in education and public administration.

A second contribution lies in the paper's implementation-oriented perspective. The proposed framework is complemented by a technical control stack, a threat model of likely failure modes, a set of governance metrics with indicative measurement approaches, and an illustrative analytical application to documented incidents. This matters because regulation-aware governance cannot remain at the level of abstract principles alone; it must be translated into controls, checkpoints, and measurable outcomes that institutions can embed in real workflows.

The paper also has limitations. First, the framework is conceptual and has not yet been validated through implementation studies, comparative experiments, or institutional pilots; the illustrative application supports analytic plausibility but not deployment effectiveness. Second, the discussion spans multiple institutional domains, which means that some controls may require contextual adaptation depending on whether the use case involves educational communication, public administration, compliance review, or rights-sensitive decision support. Third, because the regulatory landscape for AI-generated content continues to evolve, parts of the framework may need updating as disclosure, copyright, provenance, and accountability obligations become more specific across jurisdictions.

Future work should proceed in several directions. Most immediately, empirical studies are needed to test how provenance mechanisms, labels, privacy safeguards, and accountability controls perform in real institutional environments, especially under adversarial or degraded conditions such as paraphrasing, metadata loss, or harmful reuse. Three further lines of inquiry follow directly from the present analysis. First, comparative studies across jurisdictions are needed to examine how the framework's layers must be adapted to divergent regulatory regimes—for example, European, North American, and Latin American contexts—and to identify which controls are portable and which are regime-specific. Second, institutional readiness and maturity assessments should evaluate whether universities and public administrations possess the technical, procedural, and human capacity to operationalize the framework's controls, and should support the development of maturity models for AI-generated content governance. Third, user-trust research should investigate how provenance signals, disclosure labels, and visible accountability mechanisms shape the trust and behavior of students, staff, and the public, given evidence that labeling effects are uneven and context-dependent (Gamage et al., 2025; Wittenberg et al., 2025). More broadly, the next stage of research should treat AI-generated content not simply as synthetic output to be detected, but as an infrastructure of institutional risk and responsibility that must be governed across its full operational life cycle.

## Data Availability

The raw data supporting the conclusions of this article will be made available by the authors, without undue reservation.
